# Acute Cerebellitis With a Cross-Reactivity of COVID-19 and Influenza A

**DOI:** 10.7759/cureus.107692

**Published:** 2026-04-25

**Authors:** Amjad M Altunusi, Mustafa M Altoonisi, Wessam Soliman, Ali Alshehri, Mohammed Kamal

**Affiliations:** 1 Pediatric Medicine, King Salman Armed Forces Hospital, Tabuk, SAU; 2 Pediatric Intensive Care Unit, King Salman Armed Forces Hospital, Tabuk, SAU; 3 Pediatric Infectious Diseases, King Salman Armed Force Hospital, Tabuk, SAU

**Keywords:** cerebellar-ataxia, cerebillitis, cross-reactivity, influenza and covid-19 co-infection, pediatric encephalopathy

## Abstract

This is a case report of a previously healthy three-year-old male who was admitted to the emergency department (ED) of the Pediatric Intensive Care Unit in King Salman Armed Forces Hospital in Tabuk. The patient presented to the ED with an acute inability to stand, associated with two episodes of non-bilious, non-bloody vomiting and abdominal pain that began approximately four hours prior to presentation. This was preceded by a nine-day history of upper respiratory tract symptoms, for which he had received oral antibiotics. On the day of presentation, the patient developed vertigo, dysarthria, and gait instability. There was no history of fever, seizures, altered consciousness, trauma, or toxin exposure. On examination, the patient was hemodynamically stable and appeared non-toxic. Growth parameters were appropriate for age. Neurological examination revealed truncal ataxia, intention tremor, dysarthria, and bilateral hypotonia. Deep tendon reflexes were intact, with hyperreflexia noted at the knees. Cranial nerve examination was normal, and no meningeal signs were present.

Laboratory investigations were largely within normal limits, with mild elevation in inflammatory markers. Cerebrospinal fluid (CSF) analysis demonstrated mild pleocytosis, normal glucose, and mildly elevated protein. Blood, urine, and CSF cultures showed no microbial growth. CT done at presentation showed no fractures with good differentiation of gray and white matter and no central deviation. A nasopharyngeal swab was sent for viral panel, which came positive for both COVID-19 and Influenza A; thus, he was started on oseltamivir along with acyclovir, ceftriaxone, and vancomycin pending culture results. Upon stabilization of the condition and improvement of the symptoms, the patient was transferred to the floor to continue his active treatment and observation.

Acute cerebellitis should be considered in children presenting with acute ataxia, particularly following viral illness. This case highlights the potential association between SARS-CoV-2 and influenza A co-infection and cerebellar involvement. While the exact mechanism remains unclear, immune-mediated processes are likely contributory. Early recognition, appropriate investigation, and timely management are essential to ensure optimal outcomes. Further research is needed to better understand the role of viral co-infection and immune mechanisms in acute cerebellitis.

## Introduction

Acute cerebellitis (AC) is an inflammatory disorder of the cerebellum characterized by a wide spectrum of clinical presentations ranging from mild ataxia to severe neurological compromise [1]. It may occur as a primary infectious process or as a post-infectious immune-mediated condition [2]. AC is most commonly reported in children, particularly those under six years of age, and is frequently preceded by viral illnesses [3]. Several viral pathogens have been implicated, including Varicella-Zoster virus, Epstein-Barr virus, Enteroviruses, and Influenza viruses [1]. In recent years, coronavirus disease 2019 (COVID-19) has been associated with various neurological manifestations, including encephalopathy, seizures, and cerebellar ataxia [4]. Although pediatric neurological involvement remains relatively rare, the increasing number of reports suggests potential neurotropic and immune-mediated mechanisms. Cross-reactivity between viral antigens has been described, particularly between SARS-CoV-2 and Influenza A, where shared immune epitopes may lead to overlapping or exaggerated immune responses [5]. However, the clinical implications of such interactions remain uncertain. We report a case of acute cerebellitis in a child with concurrent SARS-CoV-2 and Influenza A infection, highlighting the potential role of co-infection and immune-mediated mechanisms in disease pathogenesis.

## Case presentation

A three-years-old male, previously well, was brought to Emergency Room (ER) the Pediatric Intensive Care Unit in King Salman Armed Forces Hospital in Tabuk with the complaint of inability to stand associated with two times vomiting and abdominal pain started approximately four hours prior to patient presentation preceded by history of upper respiratory tract infection nine days ago in which the patient underwent a course of amoxicillin-clavulanic acid for two days then was changed in private clinic to cefdinir due to no improvement. The data was obtained from the patient’s records that included the patient’s hard copy file, electronic file, laboratory results, and imaging. This case report discusses the patient's history, presenting complaint, hospital course, and outcomes. The management of the case is discussed, along with the rationale, to achieve a better outcome for future similar cases.

On the day of presentation, the patient woke up with abdominal pain, unable to stand with vertigo, dysarthria, and vomiting twice, mild to moderate food content projectile, no associated fever or seizures, and was then brought to our hospital. On examination, his growth parameters were within normal centiles, he did not appear dysmorphic or distressed, and was hemodynamically stable with no bruises, cyanosis, or pallor. The patient was hemodynamically stable and appeared non-toxic. No lymphadenopathies, no apparent head fractures or deformities. Deep tendon reflexes were intact, with hyperreflexia noted at the knees. Cranial nerve examination was normal, and there were no meningeal signs. No eye redness or discharge; bilateral hypotonia observed upon admission, intention tremors, inability to stand, and unsteadiness. He had no apparent spinal deformities. There was titubation and truncal ataxia; there was no stiffness, and the turning sign was negative. All reflexes were intact, with hyperreflexia noted in the knee joint bilaterally, equivocal plantar reflex bilaterally, intact abdominal reflux, and no oral ulcers. Good bilateral air entry with no added sounds. Normal first and second heart sounds, no appreciated murmurs, good capillary refill intact in central and peripheral sites, hemodynamically stable, maintaining saturation in ambient air, and the urine dipstick was unremarkable.

Venous blood gas showed pH 7.40, PO2 42 mmHg, PCO2 33 mmHg, and bicarbonate 21.4 mmol/L. Random blood glucose 5.2 mmol/L. Abdominal examination showed symmetrical contour, no abdominal distention, soft and lax, tender, no organomegaly, with adequate bowel sounds. Normal circumcised male genitalia with a normal stretched length penis. No apparent limb fractures or deformities. CT done at presentation showed no fractures with good differentiation of gray and white matter and no central deviation.

The patient was admitted as a case of cerebellitis versus cerebellar ataxia to rule out meningitis, started on ceftriaxone, vancomycin, and acyclovir on meningitic dose after opinion of the Pediatric Infectious Disease Consultant. MRI brain and MRA images showed no ischemic changes, no strokes, and no abnormal enhancements. Cerebrospinal fluid (CSF) examination revealed protein 29 g/L, glucose 3.8 mmol/L, white blood cells (WBCs) 28/µl, and all were monomorphs. A nasopharyngeal swab was sent for a viral panel, which was positive for both COVID-19 and Influenza A; thus, he was started on oseltamivir along with acyclovir, ceftriaxone, and vancomycin, pending culture results. Upon stabilization of the condition and improvement of the symptoms, the patient was transferred to the floor to continue his active treatment and observation.

Laboratory investigations were largely within normal limits, with mild elevation in inflammatory markers (Table 1). Cerebrospinal fluid (CSF) analysis demonstrated mild pleocytosis (28 cells/µL, predominantly mononuclear), normal glucose, and mildly elevated protein. Blood, urine, and CSF cultures showed no growth (Table 2).

**Table 1:** Patient's initial laboratory results ALP: Alkaline phosphatase; AST: Aspartate aminotransferase; ALT: Alanine aminotransferase; HCT: Hematocrit; MCV: Mean corpuscular volume; MCHC: Mean corpuscular hemoglobin concentration; RDW: Red cell distribution width; PLT: Platelet count; MPV: Mean platelet volume; PCT: Procalcitonin.

Variable	Value	Unit	Range
Electrolytes			
Sodium	143	mmol/L	136 - 145
Potassium	4.3	mmol/L	
Chloride	113 +	mmol/L	98 - 107
Enzymatic bicarbonate	21	mmol/L	20 - 31
Renal Function Test			
Urea nitrogen	3.00	mmol/L	1.6-4.6
Creatinine	31	µmol/L	20 - 70
Miscellaneous Chemistry			
Magnesium	0.86	mmol/L	0.61 - 0.90
Phosphate	1.35	mmol/L	1.00 - 1.90
C-reactive protein	2.70 +	mg/dl	<1
Calcium	2.39	mmol/L	2.22 - 2.47
Liver Function Test			
Total protein	66	g/L	60-80
Albumin	45 +	g/L	35 - 42
ALP	136 -	U/L	185 - 383
AST (SGOT)	30	U/L	15 - 34
ALT (SGPT)	12 -	U/L	19 - 59
Total bilirubin	4 -	µmol/L	5 - 21
Special proteins			
Anti-Streptolysis O titer	< 25	IU/ml	< 100
Hematology			
WBC	12.09	10^3^/µl	5 - 15
RBC	4.01	10^6^/µl	3.9 - 5.1
Hemoglobin	10.9 -	g/dl	11.5 - 13.5
HCT	31.5 -	%	34.0-40.0
MCV	78.6	fl	75.0 - 88.0
MCH	27.2	pg	24.0 - 30.0
MCHC	34.6	g/dl	31.0 -35.0
RDW	14.1	%	13.1 - 15.2
PLT	498 +	10^3^/µl	200 - 450
MPV	8.3	fl	7.0-13.0
PCT	0.41 +	%	0.12-0.36
Neutrophils (%)	58 +	%	15.0-35.0
Lymphocytes (%)	33 -	%	50.0-70.0
Monocytes (%)	9	%	2.0 - 10.0
Eosinophiles (%)	0 -	%	1.0 - 6.0
Basophiles (%)	0.1	%	<1 -2.0
Neutrophiles (Absolute)	7.00	10³/µL	1.5 - 8.0
Lymphocytes (Absolute)	3.97 -	10³/µL	6.0 - 9.0
Monocytes (Absolute)	1.10 +	10³/µL	0.2 - 1.0
Eosinophiles (Absolute)	0.01 -	10³/µL	0.1 - 1.0
Basophiles (Absolute)	0.01 -	10³/µL	0.02 - 0.01
Iron Profile			
Ferritin	169	ng/ml	10 - 291

**Figure 1:**
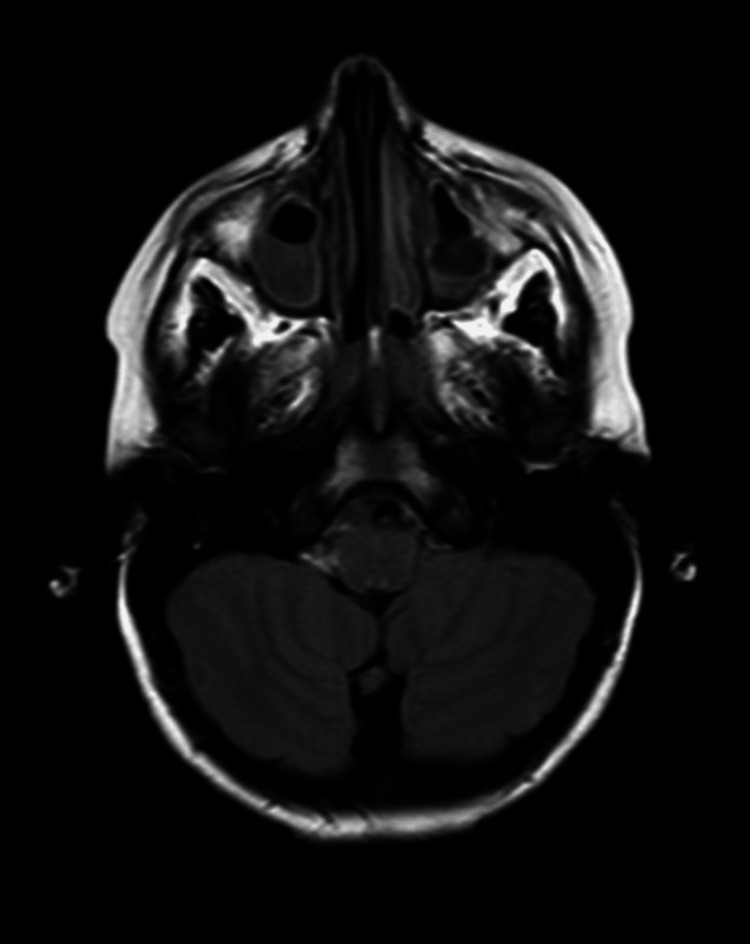
CT scan of the patient at the presentation CT scan image of the acute cerebellitis patient showed no abnormal structures, abnormal differentiation, fractures, or bleeding signs.

A computed tomography (CT) scan of the brain revealed normal gray-white differentiation with no evidence of hemorrhage, mass effect, or hydrocephalus (Figure 1). Magnetic resonance imaging (MRI) and magnetic resonance angiography (MRA) of the brain were unremarkable.

A nasopharyngeal swab for viral polymerase chain reaction (PCR) test was positive for both SARS-CoV-2 and influenza A.

**Table 2:** Patient's body fluids cultures, CSF results and respiratory panel PCR CSF: Cerebrospinal fluid; PCR: Polymerase chain reaction; Flu: Influenza.

Test	Result
Respiratory Virus Panel PCR	
COVID-19	Postitve
Flu-A1	Positive
Flu-A2	Positive
Flu-B	Negative
RSV	Negative
CSF Chemistry Analysis and Culture	
CSF glucose	3.8 mmol/L
CSF Protein	29 g/L
CSF Appearance	CLEAR
CSF Color	NO COUL
CSF WBC count	28/µl
CSF RBC count	0.0/µl
CSF Polymorph	0.0%
CSF Monomorph	100.0%
CSF Other Cells	0.0%
Body Fluids Culture	
Blood Culture	no growth
CSF Culture	no growth
Urine Culture	no growth
Throat Swab	normal flora

The patient was initially managed with intravenous ceftriaxone, vancomycin, and acyclovir. Following confirmation of influenza A infection, oseltamivir was initiated. Given persistent neurological findings and after exclusion of bacterial infection, the patient received intravenous immunoglobulin (IVIG) and pulse corticosteroid therapy. Over the following days, the patient showed significant clinical improvement, with resolution of vomiting and gradual recovery of motor function. He regained the ability to stand with minimal support and demonstrated improved coordination and speech. He was subsequently transferred to the general pediatric ward for continued care and physiotherapy. The patient was followed up one month after discharge from the hospital in the General Pediatric Clinics and was found to be in good condition with no deficits. No further complaints were raised from the family, so the patient was discharged.

## Discussion

Acute cerebellitis (AC) is an uncommon but important cause of acute ataxia in children, often occurring in the context of viral infections or post-infectious immune responses [1,2]. The clinical presentation typically includes gait instability, dysarthria, tremor, and hypotonia, with severity ranging from mild symptoms to life-threatening complications such as hydrocephalus and increased intracranial pressure [1]. The pathogenesis of AC is thought to involve autoimmune mechanisms triggered by molecular mimicry, in which antibodies generated against viral antigens cross-react with cerebellar tissue [6]. This is supported by evidence demonstrating the presence of autoantibodies in post-infectious cerebellar ataxia [6]. SARS-CoV-2 has been associated with a wide range of neurological manifestations, possibly due to direct neuroinvasion, systemic inflammation, or immune-mediated injury [4]. Influenza viruses are also well-recognized causes of central nervous system complications, including cerebellitis and encephalopathy [7,8].

In the present case, the coexistence of SARS-CoV-2 and influenza A virus raises the possibility of synergistic immune activation. Experimental studies have demonstrated cross-reactivity between T-cell responses to SARS-CoV-2 and influenza A virus, suggesting that concurrent or prior infections may modulate immune responses [5]. However, clinical evidence supporting a direct pathogenic role of cross-reactivity remains limited, and the observed findings may represent co-infection rather than true immunological interaction. Neuroimaging findings in acute cerebellitis are variable and may be normal, particularly in early or mild cases, as seen in our patient [9]. MRI is the preferred imaging modality, but it does not always reveal abnormalities, making diagnosis largely clinical.

There is no established consensus on the management of AC. Treatment is generally supportive, with consideration of antivirals, corticosteroids, and IVIG in selected cases. Previous studies have reported favorable outcomes with immunomodulatory therapy, particularly in suspected immune-mediated cases [1,10]. The prognosis of AC is generally favorable, with most patients achieving full recovery. However, some cases may develop residual neurological deficits or complications requiring neurosurgical intervention [10].

## Conclusions

Acute cerebellitis should be considered in children presenting with acute ataxia, particularly following viral illness. This case highlights the potential association between SARS-CoV-2 and influenza A virus co-infection and cerebellar involvement. While the exact mechanism remains unclear, immune-mediated processes are likely contributory. Early recognition, appropriate investigation, and timely management are essential to ensure optimal outcomes.

In clinical practice, the use of steroids, intravenous immunoglobulin, and antiviral agents yields a good outcome. Neurosurgical interventions have been performed in life-threatening situations. The complications described in the literature, such as dysmetria, involuntary tremor, and ataxia, have been seen in up to 50% of cases and are not permanent. No reports of demise were established secondary to acute cerebellitis. More studies are needed for a better approach and management for such cases. Further research is needed to better understand the role of viral co-infection and immune mechanisms in acute cerebellitis.
